# Single-Shot Dense Depth Sensing with Color Sequence Coded Fringe Pattern

**DOI:** 10.3390/s17112558

**Published:** 2017-11-06

**Authors:** Fu Li, Baoyu Zhang, Guangming Shi, Yi Niu, Ruodai Li, Lili Yang, Xuemei Xie

**Affiliations:** School of Electronic Engineering, Xidian University, Xi’an 710071, China; baoyuzhang@126.com (B.Z.); gmshi@xidian.edu.cn (G.S.); niuyi@xidian.edu.cn (Y.N.); ruodai_li@163.com (R.L.); lili.yang.research@gmail.com (L.Y.); xmxie@mail.xidian.edu.cn (X.X.)

**Keywords:** depth sensing, structured light illumination, phase unwrapping

## Abstract

A single-shot structured light method is widely used to acquire dense and accurate depth maps for dynamic scenes. In this paper, we propose a color sequence coded fringe depth sensing method. To overcome the phase unwrapping problem encountered in phase-based methods, the color-coded sequence information is embedded into the phase information. We adopt the color-encoded De Bruijn sequence to denote the period of the phase information and assign the sequence into two channels of the pattern, while the third channel is used to code the phase information. Benefiting from this coding strategy, the phase information distributed in multiple channels can improve the quality of the phase intensity by channel overlay, which results in precise phase estimation. Meanwhile, the wrapped phase period assists the sequence decoding to obtain a precise period order. To evaluate the performance of the proposed method, an experimental platform is established. Quantitative and qualitative experiments demonstrate that the proposed method generates a higher precision depth, as compared to a Kinect and larger resolution ToF (Time of Flight) camera.

## 1. Introduction

With the rapid development of computer vision and its increased use in industrial applications, depth sensing is witnessing increasing use in various fields such as biomedical testing [1,2], reverse engineering [3] and human–computer interaction. Among the numerous depth sensing methods, the structured light illumination (SLI) technique has attracted more attention owing to its advantages of fast speed, high accuracy, simplicity, and non-contact nature [4,5,6].

Based on the coding strategy, the SLI techniques [7] are generally categorized into two classes: the temporal encoding method and the spatial encoding method. The former method performs the encoding process by projecting multiple illumination patterns. Using time division multiplexing, this method can achieve a high accuracy depth map. However, it is not suitable for the dynamic scene. The representative temporal encoding methods are binary coding [8] and phase shifting [9]. The spatial method is based on the encoding of the neighborhood’s features, such as the pixel values and colors. All the coded information is integrated within one pattern, which averts the synchronization of camera and projector that is critical in the temporal encoding method. Therefore, this method is suitable for the depth sensing of moving objects. The common patterns of this method mainly include the De Bruijn coding pattern [10,11], stripe pattern [12], random pattern [13], and the M-array pattern [14]. M-array is a square pseudorandom array [15]. The structured light patterns with stripes or spots created based on a unique code are also used in the spatial encoding method. However, this method cannot be used to obtain dense depth maps because of the sparse patterns. To increase the resolution of the depth map, fringe pattern profilometry (FPP) introduces the phase measurement technique into the structured light. Two typical phase extraction methods have been widely applied to obtain the wrapped phase map in a fringe pattern: phase-shifting profilometry (PSP) [9] and Fourier transform profilometry (FTP) [16].

Because it works using a pixel-by-pixel measurement, the PSP method is insensitive to the vast variation in reflectivity on the surface of objects and can acquire a high resolution and accuracy depth [17]. In the PSP method, multiple fringe stripes with the same wavelength are usually utilized [18]. The application of pattern sequences with different periods avoids the ambiguity, which results from the fringe projection in classical phase shifting. Combined with the Gray code and the phase shifting, this approach can measure discontinuous surfaces accurately [19]. Yu et al. [20] introduce unequal period fringes to avoid the period jump error from the traditional combination of the Gray code and phase shifting. However, at least three shifted grating images are needed, which limits the application in case of dynamic scenes. A real-time measurement system based on the phase-shifting method is described in [21], which helps acquire a three dimensional (3D) shape at 30 fps, with 266 K points per frame. Zhang et al. [22] propose 3D shape measurement at 667 Hz by using a digital-light-processing (DLP) technology to switch binary structured patterns. The ambiguity introduced by high pattern frequencies has been relieved by embedding a period cue into the projected pattern [23]. Although a phase-shifting method can achieve real-time measurement by improving the frame rate, synchronization between the projector and the camera is necessary.

The unique advantage of using FTP is that it only requires a one-shot image and no synchronism for dynamic scenes. A Fourier transform (FT) is usually used to obtain a wrapped phase of single fringe patterns on smooth objects. However, it is difficult for FT to acquire the correct phase information at the edges owing to the spectral leakage in the neighborhood of discontinuities or at the areas with a large surface slope [24]. Adopting windowed Fourier transform (WFT) or wavelet transform (WT) to calculate local phase information can reduce the leakage errors [25]. 

It is critical in single-shot method to obtain absolute phase of each pixel in the modulated pattern because of periodicity of the projected pattern. Guo and Huang [26] spatially unwrapped the phase from FTP by embedding a cross-shaped marker in the single fringe pattern. The position of the marker that is utilized to calculate the absolute phase map is detected and restored before the forward. Xiao et al. [27] and Budianto et al. [28] embedded special markers and marker strips into the sinusoidal grating. However, these approaches based on markers cannot obtain the absolute phase when there is no encoded marker on the object. Meanwhile, the performance is affected in the unwrapped phase area covered by the markers. Without any additional marker, Li et al. [29] performed single-shot absolute phase recovery for the FTP method by the geometric constraints. 

A major group of approaches define color coded multi-slit or stripe patterns with a special sequence by locating intensity peaks or edges respectively in order to obtain dense reconstructions. Pagès et al. [30] designed colored stripe patterns with De Bruijn sequence where both intensity peaks and edges can be located without loss of accuracy and reducing the number of hue levels included in the pattern. Wu [31] adopted binary stripes to identify the local fringe order, while the colorful grids provides additional degree of freedom to identify the stripes. However, this encoding scheme is not available for the condition that a pure color isolated object is located in a similar color sequence period. 

This study proposes a single-shot sensing method with color sequence coded fringe to acquire precision and dense depth. Firstly, in order to design a reasonable sequence for phase period distinction, a mathematical model is established to prove the suitability of De Bruijn sequence. Secondly, two colors are used to code the De Bruijn sequence. Different from the other color-coded patterns, the phase information of each point is located at two channels, which can help us get a more precise phase distribution. Thirdly, a Gabor filter is used to extract the wrapped phase from the intensity information. Benefiting from the De Bruijn sequence, the phase unwrapping is easily achieved by color decoding. Meanwhile, based on the wrapped phase period, the error sequence order is checked and corrected by the phase neighborhoods back and forth to get a precise period order. Finally, stereo matching is accomplished to acquire the depth. Compared with the authors of [31], we used the De Brujin sequence to code the fringe and proved it, which can improve the robustness to the color scenes and different materials in a complex scene. Experiment results show that the performance of the proposed method exceeds the Kinect and ToF camera performances. 

The rest of this paper is organized as follows. The mathematical model is given in Section 2. Section 3 provides a system overview. Color sequence coded fringe pattern generation is introduced in Section 4. The phase decoding and the stereo matching are depicted in Section 5. Experiments conducted to verify the proposed method are shown in Section 6. Section 7 provides a conclusion.

## 2. Mathematical Model for Sequence Encoding

For the fringe pattern depth sensing method, the critical issue is to distinguish the period order of the wrapped phase. Here, we want to use the color information to code the sequence of the phase order. Considering that a color pattern contains three channels, the intensity values of the blue channel vary as a cosine function of a certain frequency and the remaining channels are used for sequence encoding. A sequence-coded fringe $Y_{i}$ can be defined as follows:

(1) $$Y_{i}={\left[\alpha_{i},\beta_{i},x_{i}\right]}^{T},$$

where $\alpha_{i},\beta_{i},x_{i}$ are the intensity values for the red, green, and blue channels respectively, and i is the phase period order of the current fringe. The sequence is used to code the period order. To ensure that the sequence coding only contains two colors, $\alpha_{i}$ and $\beta_{i}$ are limited in $\left\{0,x_{i}\right\}$. Considering that $x_{i}$ is the phase information, which has been set in advance and is not used as the color-coding, the distinguishable color is actually decided by

(2) $$Y_{i}={\left[\alpha_{i},\beta_{i}\right]}^{T},\;Y_{i}\in \left\{S_{0}={\left[0,x_{i}\right]}^{T},S_{1}={\left[x_{i},0\right]}^{T},S_{2}={\left[x_{i},x_{i}\right]}^{T},S_{3}={\left[0,0\right]}^{T}\right\},$$

where $S_{2}$ and $S_{3}$ are eliminated because the state $S_{2}$ results in a gray fringe and the state $S_{3}$ leads to the phase information only included in the blue channel. In a sequence coded fringe pattern, $Y_{i}$ can make full use of its neighboring fringes in the sequence to stand out from other fringes. Assuming that only the adjacent fringes $Y_{i-1}$ and $Y_{i+1}$ are combined with the current fringe to denote the subsequence $P_{i}$,

(3) $$P_{i}=\left[\begin{array}{ccc} Y_{i-1} & Y_{i} & Y_{i+1} \end{array}\right]=\left[\begin{array}{ccc} \alpha_{i-1} & \alpha_{i} & \alpha_{i+1}\\ \beta_{i-1} & \beta_{i} & \beta_{i+1} \end{array}\right].$$

The cross-correlation between any two subsequences $P_{i}$ and $P_{j}$ in the proposed pattern is calculated using the following equation:

(4) $$\mathrm{Cor}\left(P_{i},P_{j}\right)=\;\frac{P_{i}\cdot P_{j}}{j-i}=\;\frac{{\left[\begin{array}{ccc} \alpha_{i-1} & \alpha_{i} & \alpha_{i+1}\\ \beta_{i-1} & \beta_{i} & \beta_{i+1} \end{array}\right]}^{T}\left[\begin{array}{ccc} \alpha_{j-1} & \alpha_{j} & \alpha_{j+1}\\ \beta_{j-1} & \beta_{j} & \beta_{j+1} \end{array}\right]}{j-i}.$$

A subsequence is unique in the sense that a sequence means that the cross-correlation $\mathrm{Cor}\left(P_{i},P_{j}\right)$ needs to reach the minimum. In a sequence, the mathematical model of the problem can be represented as:

(5) $$\arg min\sum^{\text{​}}\mathrm{Cor}\left(P_{i},P_{j}\right)\;=\arg min\;\sum^{\text{​}}\frac{{\left[\begin{array}{ccc} \alpha_{i-1} & \alpha_{i} & \alpha_{i+1}\\ \beta_{i-1} & \beta_{i} & \beta_{i+1} \end{array}\right]}^{T}\left[\begin{array}{ccc} \alpha_{j-1} & \alpha_{j} & \alpha_{j+1}\\ \beta_{j-1} & \beta_{j} & \beta_{j+1} \end{array}\right]}{j-i}.\;s.t.\;\{\begin{array}{c} \alpha_{i},\beta_{i}\in \;\left\{0,x_{i}\right\}\\ \alpha_{j},\beta_{j}\in \;\left\{0,x_{j}\right\} \end{array}$$

Equation (5) can be simplified by the quantification to acquire an explicit solution:

(6) $$\arg min\sum^{\text{​}}\delta \left[\mathrm{Cor}\left(P_{i},P_{j}\right)\right]\;\delta \left[\mathrm{Cor}\left(P_{i},P_{j}\right)\right]=\{\begin{array}{c} 1\;\;\;\;P_{i}=P_{j}\\ 0\;\;\;\;P_{i}\neq P_{j} \end{array}.$$

The meaning of the Equation (6) is that the cross-correlation of any two subsequences $P_{i}$ and $P_{j}$ can achieve the minimum if $P_{i}\neq P_{j}$. The De Bruijn sequence with this property can achieve this aim to be used as a kind of sequence coding strategy. In this study, we want to use two colors to encode the sequence. Considering the difficulty of the sequence decoding, the length of the subsequence is three. Therefore, the circle of the De Bruijn sequence is eight. In fact, a longer length of the subsequence still meets the demand only if the subsequence can form a De Bruijn sequence.

## 3. Overview of the System

**Figure 1:**
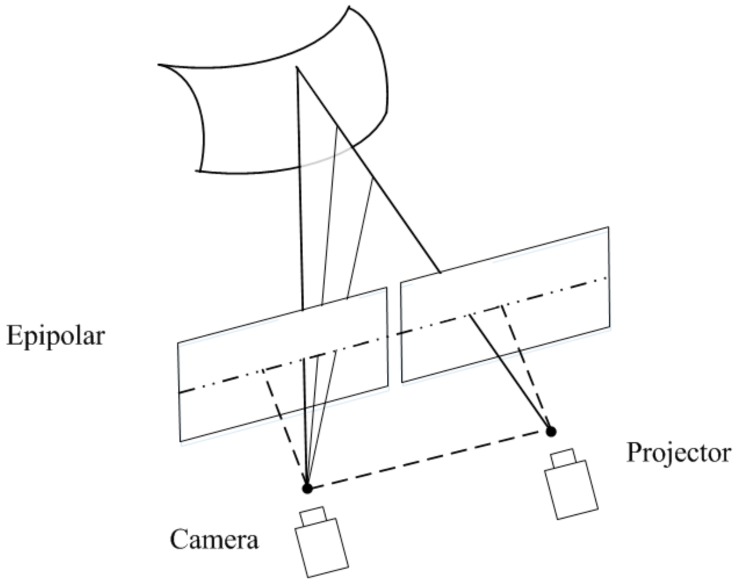
The epipolar constraint of our system.

The depth sensing system proposed in this study consists of a camera and a projector as shown in Figure 1. The dotted line and straight line mean the camera and the projector are mounted on the same horizontal plane and their optical axes are parallel. The matching points are on the same row owing to the epipolar constraint. The calibrations are accomplished in advance to obtain the intrinsic and extrinsic parameters of the camera and the projector respectively.

**Figure 2:**
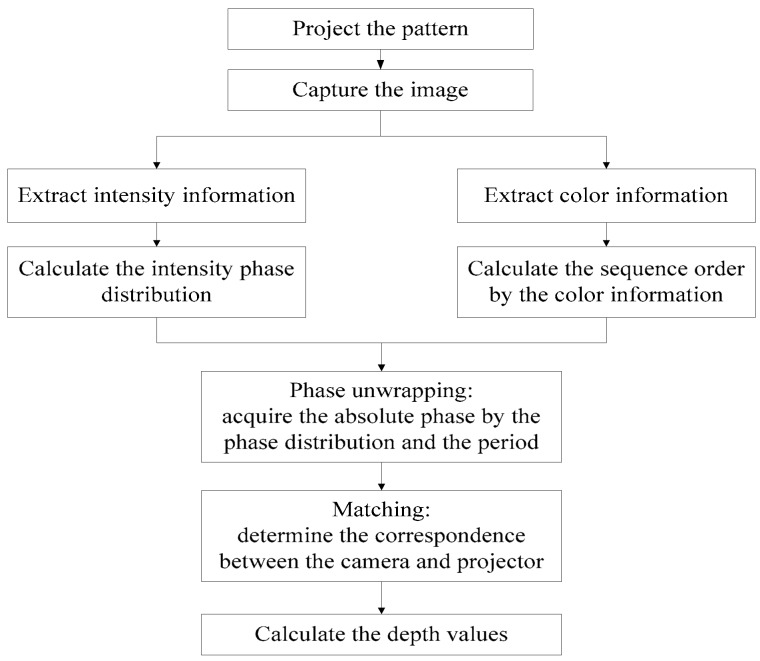
The procedure of the proposed method.

The procedure followed for the proposed method is shown in Figure 2. First, the color sequence coded fringe pattern is projected on the target object and the camera captures the modulated image. Second, the intensity information and color information are extracted from the captured image. The intensity phase distribution of the captured image is calculated from the intensity information with a Gabor filter. Third, the phase unwrapping is decoded by the De Bruijn sequence in color information. The absolute phase is acquired by the phase distribution and the period. Finally, the depth is acquired by the correspondence determination of the camera and the projector by phase stereo matching.

## 4. Color Sequence Coded Fringe Pattern

Based on the mathematical analysis in Section 2, we designed a color sequence coded fringe pattern. In this pattern, the phase encoding is adopted based on the intensity information and the De Bruijn encoding is used for the color information. The pattern generation includes two steps: in the first step, the intensity of the fringe pattern in a period varies as a cosine function of a certain frequency, which will be used for phase distribution extraction; in the second step, the De Bruijn code demonstrated by the color information is embedded into the fringe pattern to eliminate the phase ambiguity. Meanwhile, the trip point of the wrapped phase assists in overcoming the measurement sensitivity caused by the De Bruijn coding. The detailed pattern generation is as follows.

### 4.1. Phase-Coding Based on the Intensity Information

In the proposed cosine fringe pattern, the stripe direction is perpendicular to the direction of cosine coding. The intensity information I(x,y) is coded periodically in the horizontal direction. In the vertical direction, all the intensity values are the same. Assuming that the period of the cosine fringe is T, the intensity value I(x,y) in the coordinate (x,y) is defined as follows:

(7) $$I\left(x,y\right)=A+B\cos(\frac{2\pi }{T}x+\varphi_{0}),$$

where (x,y) represents the row and column coordinates in the pattern, A is a constant DC value, B is the amplitude and $\varphi_{0}$ is the initial phase of the cosine signal. In practice, we set the initial phase $\varphi_{0}$ is $\frac{\pi }{2}$. In this case, the wrapped phase is consistent with the period of the De Bruijn code, which is convenient for De Bruijn decoding. The cosine fringe pattern is shown in Figure 4.

### 4.2. De Bruijn Coding Based on the Color Information

**Figure 3:**
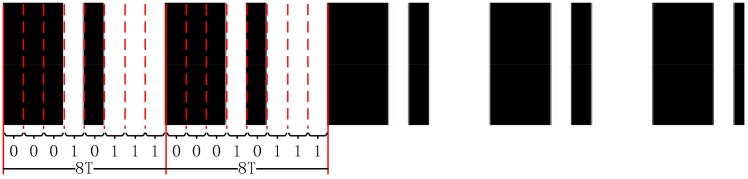
The De Bruijn coding based on the color information.

To distinguish between the period numbers of the fringe pattern, the De Bruijn sequence is adapted to generate the stripe pattern C(x,y), which only contains two values. The intensity $I_{max}$ and the intensity $I_{min}$ are labeled as 0 and 1. The De Bruijn sequence for alphabet {0,1} and 3-length subsequence is 00,010,111 as shown in Figure 3. In this stripe pattern, the width of a stripe equals the period of the fringe pattern T. Each cycle of the De Bruijn sequence consists of eight stripes. The sequence length can be set to any value like 4T, 8T, 16T, 32T… Large sequence length benefits the corresponding but the decode complexity arise significantly. We empirically choose 8T for a good balance between corresponding accuracy and computational complexity.

For color-coding, the red and green channels are adopted to represent stripe 0 and 1 respectively. Meanwhile, to ensure that the projected pattern only contains two colors, the nonzero values in the two channels must be set to be same as that in the blue channel in space. Considering that the blue channel is used for phase coding, the composite color pattern is defined as:

(8) $$\{\begin{array}{c} I_{r}\left(x,y\right)=C\left(x,y\right)I\left(x,y\right)\\ I_{g}\left(x,y\right)=\left[1-C\left(x,y\right)\right]I\left(x,y\right)\\ I_{b}\left(x,y\right)=I\left(x,y\right) \end{array}.$$

**Figure 4:**
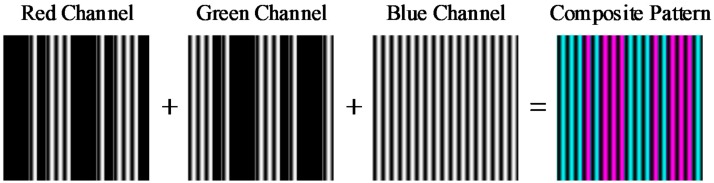
The color sequence coded fringe pattern.

Here, C(x,y) is the code value in the coordinate (x,y), 1 corresponds to the red channel and 0 corresponds to the green channel. This procedure is shown in Figure 4. In this pattern, two colors are used to code the De Bruijn sequence. Indeed, the color coded strategy is to attach the color information to the intensity information. Unlike other color-coded patterns, the phase information of each point is located at two channels: the blue channel and the red or green channel. This can help us get a more precise phase distribution. Indeed, the red channel and green channel can compose a new fringe pattern like the blue channel.

## 5. Projector–Camera Stereo Matching

After the designed pattern is projected onto the objects, the camera acquires the captured image. We first need to extract the wrapped phase from the intensity information. Then, the phase unwrapping is conducted by decoding the De Bruijn color information. Finally, the stereo matching of the projector and the camera is accomplished by the correspondence determination based on the unwrapped phase. The depth is obtained by the triangulation principle.

### 5.1. Phase Estimation

Considering that the phase information is distributed in multi channels based on the pattern design strategy, we can improve the quality of the phase information by channel overlay. The intensity information from the captured image is defined as

(9) $$\hat{I}\left(x,y\right)=\left(\hat{I_{r}}\left(x,y\right)+{\hat{I}}_{g}\left(x,y\right)+{\hat{I}}_{b}\left(x,y\right)\right)/2,$$

where $\hat{I}\left(x,y\right)$ varies cosinoidally in the horizontal direction.

In the proposed method, the Gabor filter is adapted to calculate the intensity phase distribution. The Gabor filter is a special case of the short-time FT with a local window function and specializes in the extraction of local region and frequency domain information. A two-dimension Gabor transform, a complex exponential function whose modulation kernel function is a Gaussian function, is usually used to extract the phase with a specific direction. Gabor filter is applied to the sum of the intensities of all the channels. This is because the phase information is distributed in all channels. Let G(x,y) denote the response of $\hat{I}\left(x,y\right)$ after convoluting with the 2-dimension Gabor filter, then

(10) $$G\left(x,y\right)=\left|R\left(x,y\right)\right|e^{j\left(\omega x+\varphi \left(x,y\right)\right)},$$

where R(x,y) is the amplitude of the Gabor filter response, ω and φ(x,y) represent the frequency and phase in the coordinate (x,y) respectively. The phase information φ(x,y) is calculated as follows

(11) $$\varphi \left(x,y\right)=\arctan\left[\frac{G_{r}\left(x,y\right)}{G_{i}\left(x,y\right)}\right],$$

where $G_{r}\left(x,y\right)$ and $G_{i}\left(x,y\right)$ represent the real and imaginary component of G(x,y) respectively.

In Equation (11), φ(x,y) is a periodic wrapped phase and φ(x,y)∈(−π,π). To obtain the unwrapped phase, the period of the fringe should be calculated. The unwrapped phase is defined as follows:

(12) ∅(x,y)=φ(x,y)+2nπ,

where n denotes the period number which is determined by the De Bruijn coding information.

### 5.2. Color Decoding

De Bruijn coding is based on the color information. For the image captured by camera $\hat{I}\left(x,y\right)$, the code value $\hat{C}\left(x,y\right)$ is obtained by the color component

(13) $$\hat{C}\left(x,y\right)=\{\begin{array}{c} 1\;{\hat{I}}_{r}\left(x,y\right)\geq {\hat{I}}_{g}\left(x,y\right)\\ 0\;{\hat{I}}_{r}\left(x,y\right)<{\hat{I}}_{g}\left(x,y\right) \end{array},$$

where ${\hat{I}}_{r}\left(x,y\right)$ is the red channel intensity, ${\hat{I}}_{g}\left(x,y\right)$ is the green channel intensity. However, this direct color decoding method is sensitive to the color information of the target surface. To obtain the De Bruijn code values, we adopt a voting mechanism to adjust the decoding result. De Bruijn coding is distributed in the horizontal direction. The code values in each stripe are the same. Thus, the correct code value is in a majority of the vote. After the adjustment, the code values in each stripe are made uniform and the error caused by the local color is revised.

### 5.3. Phase Unwrapping Based on De Bruijn Sequence

In the De Bruijn sequence pattern obtained after the color decoding, the two adjacent stripes cannot be distinguished from each other when their code values are the same. In terms of encoding principle, the initial phase $\varphi_{0}$ is set at $\frac{\pi }{2}$ to ensure that the wrapped phase period coincides with the period of the De Bruijn coding stripe. In case of phase unwrapping, the range of each De Bruijn stripe is obtained based on the width of the wrapped phase in the same position. Meanwhile, let us assume that the order of the stripe in a period of the De Bruijn sequence is W, where W is an integer from 1 to 8. Benefiting from the advantage of the De Bruijn sequence, one error code order can be checked by its neighborhoods. The period number n is calculated as:

(14) n=W+8*k,

where *k* is the circle number of the De Bruijn sequence.

### 5.4. Phase Based Stereo Matching 

**Figure 5:**
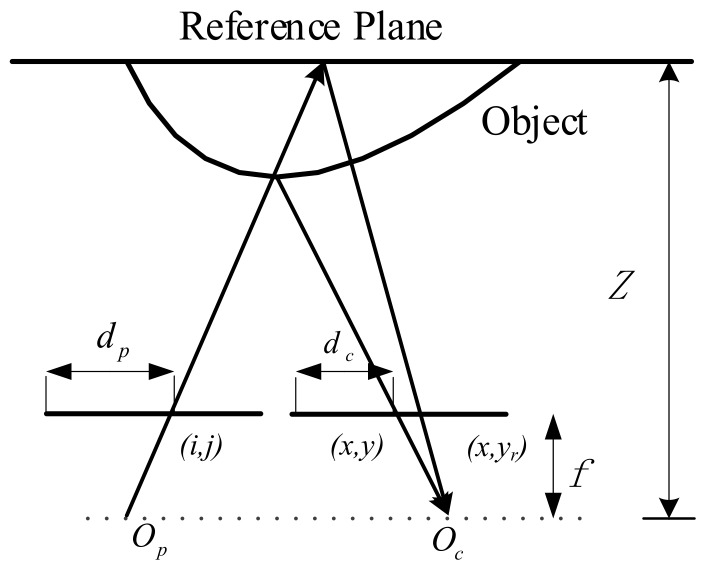
Geometry of the reference plane and the object.

In the proposed method, a reference plane technique is adopted to acquire the depth of the scene. The reference plane is a captured pattern which is projected by the projector in a given depth. The stereo matching is conducted between the reference plane and the modulated image. The geometry of the reference plane and the object as shown in Figure 5, where $O_{p}$ and $O_{c}$ are the projector optical center and the camera optical center respectively. The point (i,j) in the projected pattern is a matching point of $\left(x,y_{r}\right)$ in the camera when there is no object in front of the reference plane. In practice, the point (i,j) is a matching point of (x,y) which reflects from the point A in the object. When the epipolar constraint and the relative position between the projector and the camera, the phase of point (x,y) exhibits a shift to the left of point $\left(x,y_{r}\right)$.

Considering the similar triangles in Figure 5, the depth can be calculated by:

(15) $$Z=\frac{fB}{d},$$

where f is the focal length, B is the baseline between the camera and the projector, $d_{c}$ is the distance between current pixel and the left border in the camera image, and $d_{p}$ is the distance between the matching point and the left border in the pattern, $d=d_{c}-d_{p}$ is the disparity.

### 5.5. Simulation Experiments of the Proposed Method 

In this section, the simulation experiments are conducted to demonstrate the procedure of the proposed method. We use the 3ds Max software to simulate the SLI system. The experiments of the real scenes are given in Section 6.

**Figure 6:**
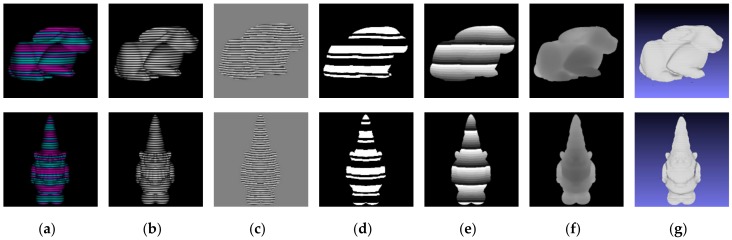
The whole procedures of the proposed method: (**a**) acquired images; (**b**) intensity information; (**c**) wrapped phase; (**d**) De Bruijn Sequence; (**e**) the unwrapped phase; (**f**) the calculated depth; (**g**) 3D reconstruction.

The whole procedure of the proposed method is shown in Figure 6. In Figure 6, (a) is the captured image; (b) illustrates the intensity information acquired by Equation (7); (c) shows the wrapped phase extracted from the intensity information by Gabor filter; (d) is the De Bruijn stripe sequence from color decoding; (e) is the unwrapped phase; (f) and (g) are the final calculated depth and 3D reconstruction of the proposed method. We can find that the proposed method can acquire a dense and accurate depth map in the simulation experiments.

**Figure 7:**
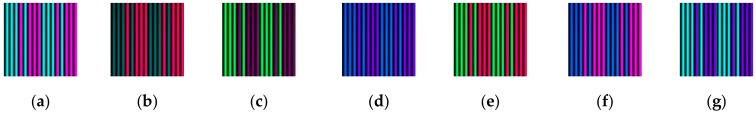
The modulated images of different color plane: (**a**) white plane; (**b**) red plane; (**c**) green plane; (**d**) blue plane; (**e**) yellow plane; (**f**) pink plane; (**g**) cyan plane.

Considering of ambient light in real scenes, experiments on different color plane are conducted to evaluate the accuracy achievable on color objects with respect to white object. The experiments are shown in Figure 7. The plane is placed at 1.0 m position from the system. A plane is fitted as the reference plane to evaluate the mean of absolute errors. In Table 1, RGB denotes the values of the red channel, green channel and the blue channel. From Table 1, we can find the errors of red plane and blue plane are a bit larger than other planes. But the results are acceptable. 

## 6. Experiment Results in Practice

**Figure 8:**
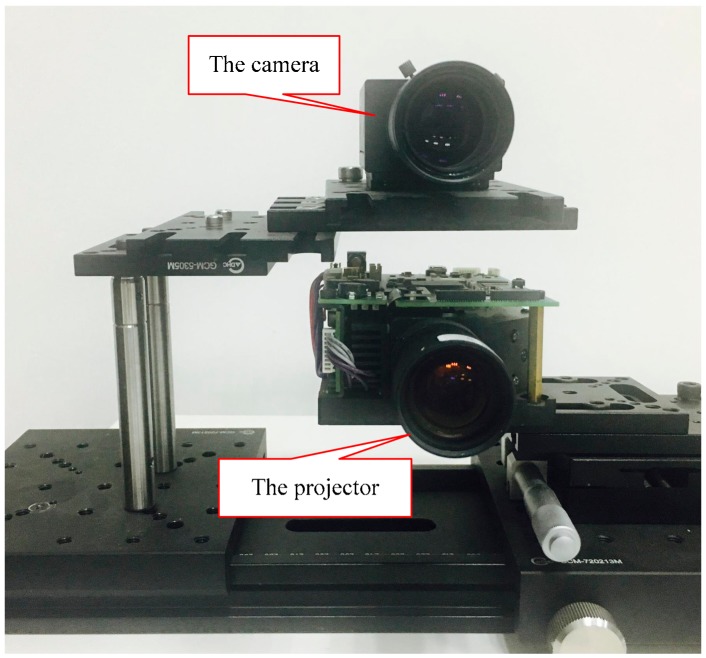
The experimental platform.

To verify the feasibility of the proposed method in practice, a series of experiments for different scenarios have been conducted. The experimental platform is established as shown in Figure 8. The camera is a FL3-U3-13E4C-C image sensor (Point Grey Flea, Richmond, BC, Canada) with 1280 × 960 resolution. The projector is DMD (Digital Micromirror Device) Light Commander instrument (Light Craft 4500 Component) (Texas Instruments, Dallas, TX, USA) with 1824 × 1140 resolution. The baseline distance between the camera and the projector is 93 mm. The optical axes of the camera and the projector are parallel. In our experiment, we try our best to reduce the influence of the disalignment. In the designed pattern, the period of the fringe is 21 pixels and the period of the De Bruijn sequence is 8 stripes. The experimental platform is aligned vertically in advance so that the epipolar lines are along the vertical direction are based on the epipolar constraint. The projector-camera platform is calibrated by the plane-based calibration method [32]. This method is implemented as an extension of the Bouguet Camera calibration toolbox [33]. The intrinsic and extrinsic parameters are shown in Table 2. The point clouds of the recovery scenes are reconstructed by MeshLab software [34]. Quantitative and qualitative experiments are employed to evaluate the performance of the proposed method.

### 6.1. Quantitative Analysis

**Figure 9:**
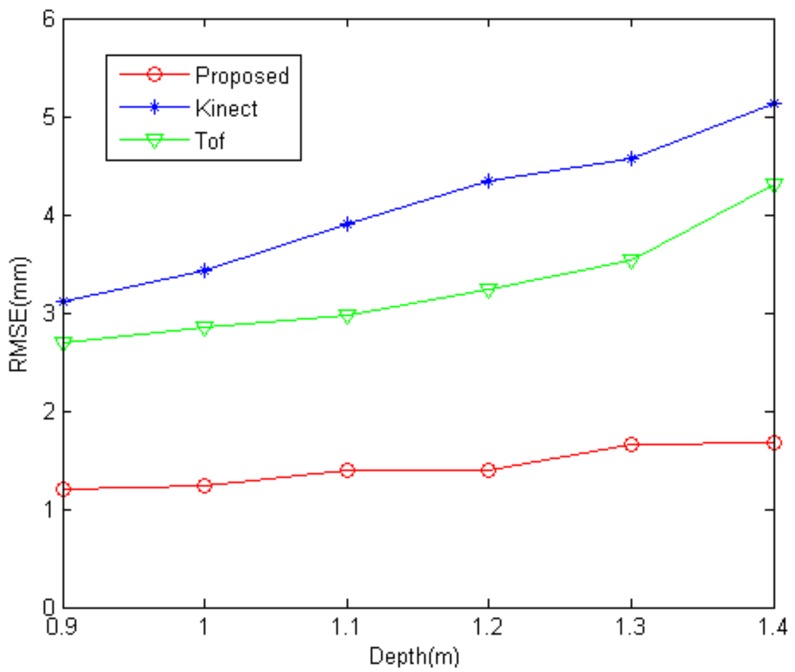
Root mean square error (RMSE) at different depths for the three different methods.

Firstly, we calculate the root mean square error (RMSE) for a series of planes placed at different depths ranging from 0.9 to 1.4 m. A Kinect and a ToF camera SwissRanger 4000 (Mesa Imaging, Zürich, Switzerland) are used as the competitors. Each position of the plane is measured more than 10 times. The quantitative results of the comparative experiment are shown in Figure 9 where the measurement unit is mm. The tendency of the RMSE adheres to the rule that the measurement precision decreases with increasing distance. From this figure, it can be observed that the performance of our proposed method is better than that of Kinect and ToF camera. 

**Figure 10:**
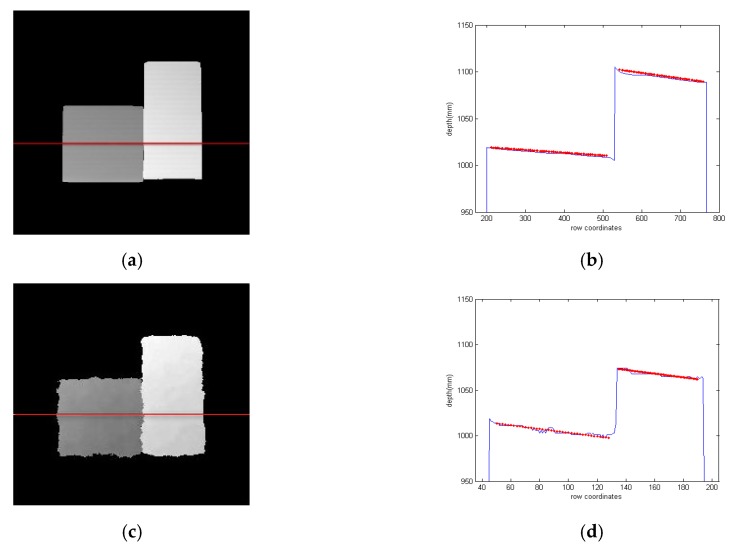

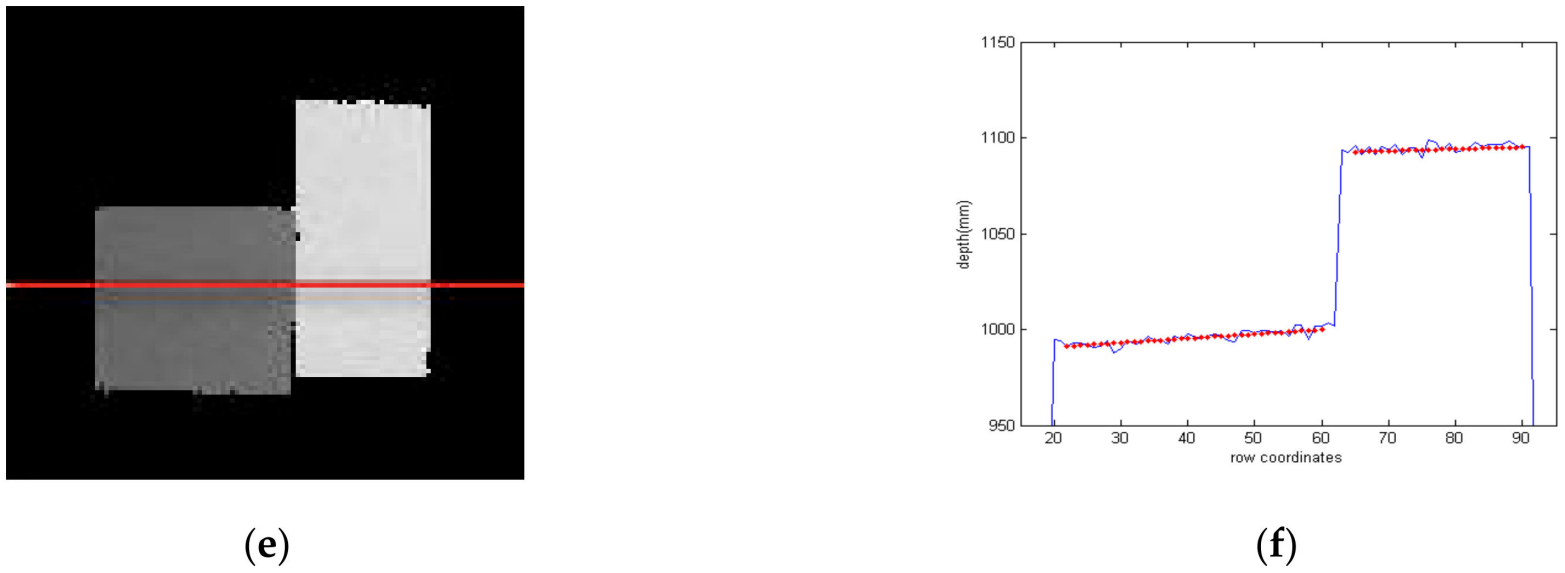
The depth of the discontinuous surface: (**a**) depth maps acquired by the proposed method; (**b**) cross-section plot of the proposed method; (**c**) depth maps acquired by Kinect; (**d**) cross-section plot of Kinect; (**e**) depth maps acquired by ToF; (**f**) cross-section plot of ToF.

In addition, the measurement of the discontinuous surface is used as another metric to evaluate the performance of our method. In this scene, a cuboid next to a cube is placed at a different distance from the camera so that the junction of two objects forms a discontinuous surface. We try our best to adjust the three systems to have the same depth to the object and adopt the relevant errors as the metric to replace the absolute errors. The performance of our method is shown in Figure 10a,b. The results of Kinect (Figure 10c,d) and ToF (Figure 10e,f) are also used as the benchmarks to evaluate the performance. Figure 10b,d,f are the cross-section-plot results for the same position in Figure 10a,c,e respectively. The red dotted lines are the actual depth obtained by the Least Squares Fitting method in the position. Table 3 provides the mean of absolute errors for the three competitors. We can find that our method generates smaller errors than Kinect and ToF camera, which can validate the precision of the proposed method.

### 6.2. Qualitative Results

**Figure 11:**
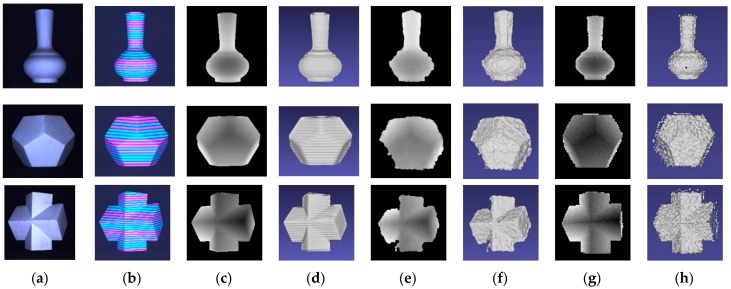
The depth of the geometries: (**a**) real scenes; (**b**) acquired images; (**c**) depth maps acquired by the proposed method; (**d**) 3D reconstruction of the proposed method; (**e**) depth maps acquired by Kinect; (**f**) 3D reconstruction of Kinect; (**g**) depth maps acquired by ToF; (**h**) 3D reconstruction of ToF.

For visualization of the results obtained by the proposed method, especially the recovery of edges of the object, some plaster geometries are placed at a distance of about 1 m from our platform. The actual scene and the acquired images are shown in Figure 11a,b. In Figure 11, the bottleneck of the vase is concave downward and the body of the vase is an upward convex. The last two geometries contain smooth areas but with sharp edges. The depth map and the cloud point of the proposed method are shown in Figure 11c,d respectively. The results of Kinect and ToF camera are given in Figure 10e,f and Figure 11c,d . Benefitting from the accurate phase unwrapping procedure, our proposed method can not only recover the depth of the smooth surfaces and clear edges but also acquire the curved areas such as the surface of the vase. In case of recovery using Kinect and ToF camera, the edge is blurred and the surface is coarse because of the low precision and resolution.

**Figure 12:**
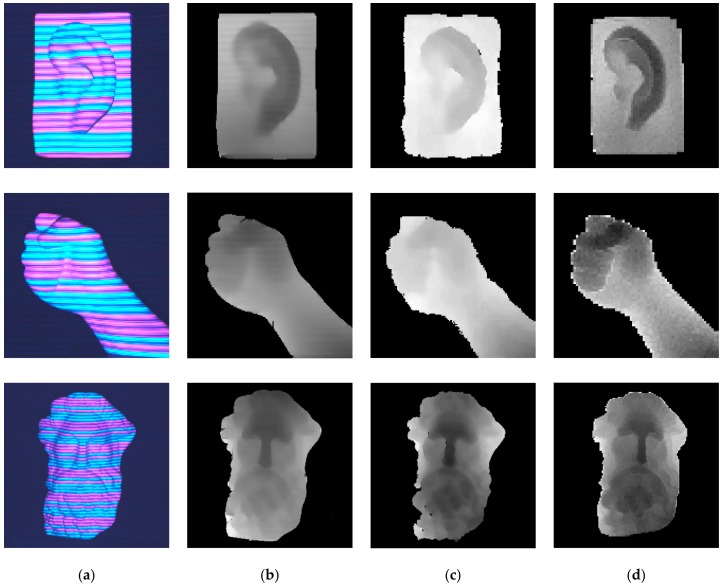
The depth of the body geometries: (**a**) acquired images; (**b**) depth maps acquired by the proposed method; (**c**) depth maps acquired by Kinect; (**d**) depth maps acquired by ToF.

In addition, some sculptures of the human body parts are selected to demonstrate the feasibility in case of variations in the surface texture. The depth maps acquired by the proposed method, Kinect and ToF are shown in Figure 12b–d respectively. From the depth maps, we can see that some details such as the recovery of ear and figure are lost and the profiles are blurred in the depth map obtained by Kinect. Although the objects recovered by ToF camera are clear, the resolution of ToF camera is only 176 × 144. The granular effect of ToF camera results is high which affects the 3D reconstruction significantly. Different from the blurring and granular visualization, the depth maps are clear in our proposed method, especially for the hair and mustache of the man sculpture. This experiment can reflect the high accuracy of our method. In this experiment, the period of 21 pixel is kept the same in all of the measurements. The appearance of period of fringes in Figure 12a,b seems different with (c) because we zoom the objects into different ratio for better exhibition of the result.

**Figure 13:**
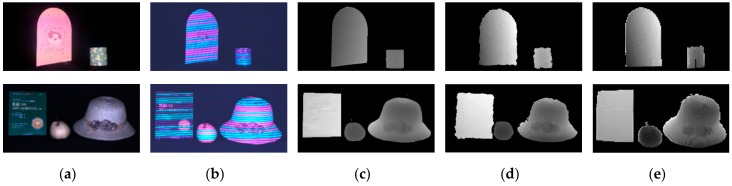
The depth of the color discontinuous scenes: (**a**) real scenes; (**b**) acquired images; (**c**) depth maps for the proposed method; (**d**) depth maps obtained from Kinect; (**e**) depth maps obtained from ToF.

Color and complex scenes present a challenge because the surface color of objects may lead to errors in the color decoding process. Moreover, the optical absorption varies with different materials, which results in sensitive sensing. To validate that the proposed method is robust to the color scenes and different materials in a complex scene, we select two scenes with multiple objects and rich colors as shown in Figure 13. The surface of the bookrack, pot, and book are smooth and made of specular material while the surface of the pear and straw hat is diffuse and made with a rough material. The results of Kinect and ToF are also shown in this Figure. The pink and cyan colors in the first scene are similar to those in the proposed pattern. However, benefiting from our pattern design strategy, the details of the depths maps are clear and dense, which can prove that the proposed method outperforms the Kinect and ToF cameras, both in precision and resolution.

## 7. Conclusions

In this paper, a single-shot sensing method with color sequence coded fringe is proposed to acquire precise and dense depth. Color coded sequence information is embedded into the phase information to relieve the phase unwrapping. On the one hand, the phase information of each point is located at multiple channels, which can help us get a more precise phase distribution. On the other hand, a wrapped phase period assists the sequence decoding to get a precise period order. We have established a theoretical model to prove the suitability of the De Bruijn sequence and constructed an experimental platform to verify the performance of the proposed method. The results show that our method can demonstrate excellent performance terms of precision, as well as resolution, as compared to off-the-shelf devices.

## Figures and Tables

**Table 1:** RGB value and the mean of absolute errors for our method on different color plane.

	White	Red	Green	Blue	Yellow	Pink	Cyan
RGB	255,255,255	255,0,0	0,255,0	0,0,255	255,255,0	255,0,255	0,255,255
Errors (unit: mm)	0.42	0.83	0.84	0.41	0.46	0.55	0.56

**Table 2:** The intrinsic and extrinsic parameters of camera and projectors (unit: pixel).

	Camera	Projector
focal length	2312.5320	2227.9948
principal points	${\left[\begin{array}{cc} 1027.9968 & 1014.5375 \end{array}\right]}^{T}$	${\left[\begin{array}{cc} 467.2511 & 1055.7085 \end{array}\right]}^{T}$
tangential distortions	$\left[\begin{array}{ccc} 1.5906 & -101.00 & -21.8810 \end{array}\right]$	$\left[\begin{array}{ccc} -1.5906 & 101.00 & 21.8810 \end{array}\right]$
radial distortions	$\left[\begin{array}{ccc} 0.9999 & -0.0051 & -0.0090\\ 0.0045 & 0.9984 & -0.0566\\ 0.0093 & 0.0565 & 0.9984 \end{array}\right]$	$\left[\begin{array}{ccc} 0.9999 & 0.0045 & 0.0093\\ -0.0051 & 0.9984 & 0.0565\\ -0.0090 & -0.0566 & 0.9984 \end{array}\right]$

**Table 3:** The mean of absolute errors for the three methods on the discontinuous surface (unit: mm).

	The Proposed Method	Kinect	ToF Camera
The cube	0.8366	1.4665	1.8699
The cuboid	1.3345	1.4599	1.9811

## References

[1] Lilley F., Lalor M.J., Burton D.R. (2000). Robust fringe analysis system for human body shape measurement. OPTICE.

[2] Genovese K., Pappalettere C. (2006). Whole 3D shape reconstruction of vascular segments under pressure via fringe projection techniques. Opt. Lasers Eng..

[3] Lin C.-H., He H.-T., Guo H.-W., Chen M.-Y., Shi X., Yu T. (2005). Fringe projection measurement system in reverse engineering. J. Shanghai Univ..

[4] Zhang Z., Jing Z., Wang Z., Kuang D. (2012). Comparison of fourier transform, windowed fourier transform, and wavelet transform methods for phase calculation at discontinuities in fringe projection profilometry. Opt. Lasers Eng..

[5] Gorthi S.S., Rastogi P. (2010). Fringe projection techniques: Whither we are?. Opt. Lasers Eng..

[6] Quan C., Chen W., Tay C.J. (2010). Phase-retrieval techniques in fringe-projection profilometry. Opt. Lasers Eng..

[7] Salvi J., Fernandez S., Pribanic T., Llado X. (2010). A state of the art in structured light patterns for surface profilometry. Pattern Recognit..

[8] Posdamer J.L., Altschuler M. (1982). Surface measurement by space-encoded projected beam systems. Comput. Graph. Image Process..

[9] Gupta M., Nayar S.K. Micro phase shifting. Proceedings of the IEEE Conference on Computer Vision and Pattern Recognition.

[10] Tuliani J. (2001). De bruijn sequences with efficient decoding algorithms. Discret. Math..

[11] Monks T., Carter J., Shadle C. Colour-encoded structured light for digitisation of real-time 3D data. Proceedings of the International Conference on Image Processing and its Applications.

[12] Li Q., Li F., Shi G., Qi F., Shi Y., Gao S. Dense depth acquisition via one-shot stripe structured light. Proceedings of the Conference on Visual Communications and Image Processing (VCIP).

[13] Yang Z., Xiong Z., Zhang Y., Wang J., Wu F. Depth acquisition from density modulated binary patterns. Proceedings of the IEEE Conference on Computer Vision and Pattern Recognition.

[14] Chen S., Li Y., Zhang J. Realtime structured light vision with the principle of unique color codes. Proceedings of the IEEE International Conference on Robotics and Automation.

[15] Morano R.A., Ozturk C., Conn R., Dubin S., Zietz S., Nissanov J. (1998). Structured light using pseudorandom codes. IEEE Trans. Pattern Anal. Mach. Intell..

[16] Takeda M., Mutoh K. (1983). Fourier transform profilometry for the automatic measurement of 3-D object shapes. Appl. Opt..

[17] Song L., Chang Y., Xi J., Guo Q., Zhu X., Li X. (2015). Phase unwrapping method based on multiple fringe patterns without use of equivalent wavelengths. Opt. Commun..

[18] Lohry W., Chen V., Zhang S. (2014). Absolute three-dimensional shape measurement using coded fringe patterns without phase unwrapping or projector calibration. Opt. Express.

[19] Lilienblum E., Michaelis B. (2007). Optical 3D surface reconstruction by a multi-period phase shift method. JCP.

[20] Yu S., Zhang J., Yu X., Sun X., Wu H. (2016). Unequal-period combination approach of gray code and phase-shifting for 3-D visual measurement. Opt. Commun..

[21] Zhang S., Yau S.-T. (2006). High-resolution, real-time 3D absolute coordinate measurement based on a phase-shifting method. Opt. Express.

[22] Zhang S., Van Der Weide D., Oliver J. (2010). Superfast phase-shifting method for 3-D shape measurement. Opt. Express.

[23] Wang Y., Liu K., Hao Q., Lau D.L., Hassebrook L.G. (2011). Period coded phase shifting strategy for real–time 3-D structured light illumination. IEEE Trans. Image Process..

[24] Su X., Chen W. (2001). Fourier transform profilometry: A review. Opt. Lasers Eng..

[25] Berryman F., Pynsent P., Cubillo J. (2004). The effect of windowing in fourier transform profilometry applied to noisy images. Opt. Lasers Eng..

[26] Guo H., Huang P.S. (2009). Absolute phase technique for the fourier transform method. OPTICE.

[27] Xiao Y.S., Xian-Yu S.U., Zhang Q.C., Ze-Ren L.I. (2007). 3-D profilometry for the impact process with marked fringes tracking. Opto-Electron. Eng..

[28] Budianto B., Lun P.K., Hsung T.C. (2014). Marker encoded fringe projection profilometry for efficient 3D model acquisition. Appl. Opt..

[29] Li B., An Y., Zhang S. (2016). Single-shot absolute 3D shape measurement with fourier transform profilometry. Appl. Opt..

[30] Pagès J., Salvi J., Collewet C., Forest J. (2005). Optimised de bruijn patterns for one-shot shape acquisition. Image Vis. Comput..

[31] Su W.H. (2008). Projected fringe profilometry using the area-encoded algorithm for spatially isolated and dynamic objects. Opt. Express.

[32] Falcao G., Hurtos N., Massich J. (2008). Plane-based calibration of a projector-camera system. Vibot Master.

[33] Camera Calibration Toolbox for Matlab. http://www.vision.caltech.edu/bouguetj/calib_doc/index.html.

[34] Meshlab Software. http://www.meshlab.net/.

